# Comparison of the Nutritional Status of Overseas Refugee Children with Low Income Children in Washington State

**DOI:** 10.1371/journal.pone.0147854

**Published:** 2016-01-25

**Authors:** Elizabeth E. Dawson-Hahn, Suzinne Pak-Gorstein, Andrea J. Hoopes, Jasmine Matheson

**Affiliations:** 1 Department of Pediatrics, University of Washington, Seattle, Washington, United States of America; 2 Center for Child Health, Behavior and Development, Seattle Children’s Research Institute, Seattle, Washington, United States of America; 3 Department of Global Health, University of Washington, Seattle, Washington, United States of America; 4 Department of Pediatrics, University of Colorado School of Medicine, Aurora, Colorado, United States of America; 5 Refugee Health Program, Office of Communicable Disease Epidemiology, Washington State Department of Health, Shoreline, Washington, United States of America; University of São Paulo, BRAZIL

## Abstract

**Introduction:**

The extent that the dual burden of undernutrition and overnutrition affects refugee children before resettlement in the US is not well described.

**Objective:**

To describe the prevalence of wasting, stunting, overweight, and obesity among refugee children ages 0–10 years at their overseas medical screening examination prior to resettlement in Washington State (WA), and to compare the nutritional status of refugee children with that of low-income children in WA.

**Methods:**

We analyzed anthropometric measurements of 1047 refugee children ages 0–10 years old to assess their nutritional status at the overseas medical screening examination prior to resettlement in WA from July 2012—June 2014. The prevalence estimates of the nutritional status categories were compared by country of origin. In addition, the nutritional status of refugee children age 0–5 years old were compared to that of low-income children in WA from the Center for Disease Control and Prevention’s Pediatric Nutrition Surveillance System.

**Results:**

A total of 982 children were eligible for the study, with the majority (65%) from Somalia, Iraq and Burma. Overall, nearly one-half of all refugee children had at least one form of malnutrition (44.9%). Refugee children ages 0–10 years were affected by wasting (17.3%), stunting (20.1%), overweight (7.6%) and obesity (5.9%). Among children 0–5 years old, refugee children had a significantly higher prevalence of wasting (14.3% versus 1.9%, p<0.001) and stunting (21.3% versus 5.5%, p<0.001), and a lower prevalence of obesity (6.2% versus 12.9%, p<0.001) than low-income children in WA.

**Conclusion:**

The dual burden of under- and over-nutrition among incoming refugee children as well as their overall difference in prevalence of nutritional status categories compared to low-income children in WA provides evidence for the importance of tailored interventions to address the nutritional needs of refugee children.

## Introduction

Refugee children represent an expanding vulnerable population in the United States (US) at high risk for health and nutritional disparities. The number of refugees in the world continues to increase with 2014 estimates of 14.4 million individuals meeting the United Nations High Commission for Refugees (UNHCR) definition, representing the highest numbers of refugees reported since 1995 [1]. Approximately half of the world’s refugee population are children under the age of 18 years [1]. In the US, children ages 0–17 years old represented one-third of the 69,909 refugees resettled in the year 2013 [2, 3]. Washington (WA) was among the top 10 resettlement states in 2012, resettling a total of 2,165 refugees, and similarly one-third were children ages 0–17 years old [4].

Refugee children arrive in the US from countries with a high burden of undernutrition, infectious disease, and poverty [5]. Undernutrition increases a child’s risk of morbidity and mortality associated with infectious disease, and poor cognitive and developmental outcomes [6]. Undernutrition is not, however, the only form of malnutrition affecting children worldwide. Children, like adults, are experiencing an increasing prevalence of overnutrition (overweight and obesity) [7]. While high income countries have the greatest rates of overweight children, the majority of the world’s overweight children now live in low and middle income countries [6]. Childhood overnutrition leads to adult obesity and the associated long term health risks of hypertension, diabetes mellitus type II, liver and gall bladder disease, cancers, and depression [8]. Refugee children may originate from countries with a prevalence of both undernutrition and overnutrition.

Overseas nutritional surveillance of refugee children has primarily focused on the burden of undernutrition with limited reporting on overnutrition [9]. One recent exception is a 2014 Centers for Disease Control and Prevention (CDC) report that found Syrian refugee children were on average more overweight than acutely malnourished [10]. Published reports on refugee children from other countries are lacking, however. Young children under 5 years old are frequently the focus of overseas nutritional surveillance programs, however much less is known regarding older children. Children ages 5–10 years old have the potential for catch-up growth [11], and both under- and overnutrition have long term implications [6]. After resettlement, an increasing prevalence of overnutrition has been reported among refugee children in the US and Australia [12–14]. It is essential, therefore, to evaluate the burden of both under- and over-nutrition among a wide age range of incoming refugee children in order to provide appropriate support before and after resettlement.

Our study aimed to describe the prevalence of wasting, stunting, overweight, and obesity at the overseas medical screening examination for refugee children 0–10 years old resettling in WA. We also aimed to compare the nutritional status categories of incoming refugee children 0–5 years old to WA low-income children included in the CDC’s Pediatric Nutrition Surveillance System (PedNSS).

## Methods

Before refugees leave their country of departure for the US, they have an overseas medical screening examination (OME) conducted by a physician contracted by the US State Department [15]. After arrival in the US, the refugees undergo a domestic medical screening examination (DME) at their local public health department or in a primary care clinic contracted by the state department of health [16]. The CDC OME technical instructions include conducting a physical examination and anthropometric measurements, and the CDC DME guidelines include nutrition and growth [17].

We examined the OME records of all refugee children resettled in WA from July 1, 2012 to June 30, 2014 from the WA Department of Health via the CDC’s Electronic Disease Notification system. Weight and height/length measurements were obtained at their OME conducted by medical professionals as a routine component of the physical examination [15, 18, 19]. Following World Health Organization (WHO) and CDC guidelines for measuring anthropometry, standing height was measured for children greater than or equal to two years old and supine length for children under two years old [20]. Inclusion criteria were as follows: 1) data available for both weight and height/length and 2) children aged 10 years and younger at the time of their OME. Children with growth measurements that were not physiologically plausible were removed including those with weight-for-length, body-mass-index (BMI) or height/length-for-age z-scores greater than 5 standard deviations above or below the mean. The Washington State Institutional Review Board and Seattle Children’s Institutional Review Board each independently approved this study. All data was de-identified for analysis.

Data from refugee children were analyzed in aggregate as well as by country of origin for the top three countries of origin: Iraq, Somalia, and Burma, representing 65% of the sample. A subgroup of refugee children under 5 years old were compared with the CDC’s 2011 WA PedNSS data. The PedNSS is a national CDC nutritional surveillance system of low income children across the US, and data is reported both for the entire US and state-by-state [21]. The majority of the PedNSS data are from participants in the Special Supplemental Nutrition Program for Women, Infants, and Children (WIC); and the remaining data are from the Medicaid Early and Periodic Screening, Diagnosis, and Treatment (EPSDT) program and the Title V Maternal and Child Health (MCH) program. The 2011 WA PedNSS data included growth indicators for 222,048 low-income children ages 0–5 years old.

Children in our study were categorized into one of four weight-based nutritional status categories: wasting, healthy weight, overweight or obesity, based on the child’s age and gender appropriate weight-for-height. Children with a low weight-for-height/length proportions are classified into the wasting category. This is often associated with a period of acute starvation or severe disease [20]. Healthy weight children are those that fall in the range between acute wasting and overweight. Children with a high weight-for-height/length proportion are classified as being overweight, or with higher values as being obese, which is associated with a higher energy intake than is necessary for their stature [20]. In addition, children in these four weight-based nutritional status categories were also classified into a fifth height-based nutritional status category of stunting if they fit the criteria, indicated by a low height/length-for-age. These children typically fail to reach their linear growth potential due to a prolonged period of suboptimal health and nutrition [20].

The nutritional status categories are based on anthropometric definitions from the World Health Organization growth standard (WHO 2006) definitions for children 0–1.99 years of age and from the Centers for Disease Control and Prevention (CDC 2000) definitions for children 2–10 years old, which align with the definitions utilized by the PedNSS (Table 1) [20, 22, 23], and are the recommended definitions by the CDC for children in the US [24]. Prevalence estimates were also calculated utilizing the WHO 2006 guidelines for all refugee children ages 0–10 years old since these definitions are commonly used globally and are based on the Multicenter Reference Group Study [20]. The WHO anthropometric definitions are included as S1 Table.

**Table 1:** Nutritional status category anthropometric definitions based on WHO 2006 and CDC 2000 definitions.

Nutritional status category	Age 0–1.99 years^∫^	2–10 years^€^
Stunting*	Length-for-age ≤ 2.3^rd^ percentile	Height-for-age < 5^th^ percentile
**Wasting**	Weight-for-length ≤ 2.3^rd^ percentile	BMI < 5^th^ percentile
**Healthy weight**	Weight-for-length > 2.3^rd^ percentile and < 97.7^th^ percentile	BMI ≥ 5^th^ percentile and ≤ 85^th^ percentile
**Overweight**	Not included^α^	BMI > 85^th^ percentile and < 95^th^ percentile
**Obesity**	Weight-for-length ≥ 97.7^th^ percentile	BMI ≥ 95^th^ percentile

The 2.3^rd^ percentile is equal to z-score of -2.00, 5^th^ percentile is equal to z-score of -1.64, 85^th^ percentile is equal to a z-score of 1.04, 95^th^ percentile is equal to a z-score of 1.64, and 97.7^th^ percentile is equal to a z-score of 2.00.

^**∫**^ Based on WHO 2006 definitions.[20]

^€^Based on CDC 2000 definitions. [22]

*Children classified into one of the four weight-for-height categories, could also be classified into the height/length-for-age category of stunting.

^α^An overweight category is not included in the PedNSS data for this age group.

The WHO Anthro and WHO Anthroplus Macros [25, 26] in Stata 13.0 (College Station, TX: StataCorp LP) were used to calculate the anthropometric indices based on WHO 2006 criteria. Stata 13.0 (College Station, TX: StataCorp LP) and the program *zanthro* [27], which is based on the CDC 2000 reference values, were used to calculate the height/length-for-age and BMI z-scores for children 2–10 years old [22]. Chi square tests were used to compare the nutritional status categories by countries of origin for the primary analysis of refugee children 0–10 years of age. A subgroup analysis was conducted using binomial tests to compare the prevalence of nutritional status categories of refugee children under 5 years old with the prevalence of nutritional status categories of low-income children in the WA PedNSS data.

## Results

### Study population

A total of 1,047 refugee children ages 0–10 years old were resettled in WA from July 1, 2012 to June 30, 2014. Within this sample, 41 children did not have weight and height data available from their OME, and 24 children with reported weight-for-length, BMI or height/length-for-age z-scores greater than 5 standard deviations above or below the mean were excluded. Therefore, 982 children were included in these analyses. The median age of the refugee children was 4.9 years old (interquartile range: 2.5–7.3 years), 46.7% were female, and 51.1% were under 5 years old. Children from 35 countries of origin were resettled in WA during the study period, and the top three countries of origin represented 65.2% of all refugee children resettled in WA: Iraq (n = 267, 27.2% of the sample), Somalia (n = 219, 22.3%), and Burma (n = 154, 15.7%).

### Nutritional status of refugee children

Overall nearly half of all children 0–10 years of age had at least one form of malnutrition (44.9%, n = 441). The prevalence of wasting among these children was 17.3%, stunting was 20.1%, overweight was 7.6%, and obesity was 5.9%. When divided by age the prevalence estimates differed (Table 2) with the most notable difference being that older children, 5–10 years of age, possessed a higher prevalence of wasting than the younger refugee children (20.4% versus 14.3%).

**Table 2:** Prevalence estimates for the nutritional status categories of refugee children at the overseas screening medical examination overall, % (95% CI).

Nutritional status category	All Countries		
	<5 years	5–10 years	All Ages
	n = 502	n = 480	n = 982
Stunting*	21.3 (17.7–24.9)	18.8 (15.2–22.3)	20.1 (17.6–22.6)
**Wasting**	14.3 (11.3–17.4)	20.4 (16.8–24.0)	17.3 (14.9–19.7)
**Healthy weight**	70.9 (66.9–74.9)	67.3 (63.1–71.5)	69.1 (66.3–72.0)
**Overweight**	8.6 (6.1–11.0)	6.7 (4.4–8.9)	7.6 (6.0–9.3)
**Obesity**	6.2 (4.1–8.3)	5.6 (3.6–7.7)	5.9 (4.4–7.4)

* For the overall sample there were 37 children with stunting and wasting, and 17 children with stunting and overweight or obesity. The remaining children with stunting are also classified in the healthy weight category.

The prevalence of the nutritional status categories varied among the top three countries of origin (Table 3). Somali children age 0–10 years old had the highest prevalence of wasting (29.2%) compared to the Iraqi and Burmese children, (16.6% and 8.4%, respectively, p<0.001). Burmese children age 0–10 years old had the highest prevalence of stunting (38.3%) compared to the Somali and Iraqi children (p<0.001). Children from Iraq had the highest prevalence of overweight (9.8%) and obesity (9.4%) compared to the Somali and Burmese children (p<0.001).

**Table 3:** Prevalence estimates for the nutritional status categories of refugee children at the overseas screening medical examination by country of origin, % (95% CI).

Nutritional status category	Iraq			Somalia			Burma		
	< 5 years	5–10 years	All ages	< 5 years	5–10 years	All ages	< 5 years	5–10 years	All ages
	n = 147	n = 118	n = 265	n = 99	n = 120	n = 219	n = 84	n = 70	n = 154
Stunting*	10.2 (5.3–15.2)	4.2 (0.5–7.9)	7.5 (4.3–10.7)^€^	26.2 (17.4–35.1)	15.8 (9.2–22.5)	20.5 (15.2–25.1)^€^	29.8(19.8–39.7)	48.6 (36.6–60.6)	38.3 (30.5–46.1)^€^
**Wasting**	19.7 (13.2–26.2)	12.7 (6.6–18.8)	16.6 (12.1–21.1)^€^	23.2 (14.8–31.7)	34.2 (25.6–42.8)	29.2 (23.2–35.3)^€^	4.7(0–9.4)	12.9 (4.8–20.9)	8.4 (4.0–12.9)^€^
**Healthy weight**	64.6 (56.8–72.4)	62.6 (54.7–72.4)	64.2 (58.3–70.0)	58.6 (48.7–68.5)	59.2 (50.2–68.1)	58.9 (52.3–65.6)	84.5(76.6–92.4)	77.1 (67.1–87.2)	81.2 (74.9–87.4)
**Overweight**	8.8 (4.2–13.5)	11.0 (5.3–16.7)	9.8 (6.2–13.4)	8.1 (2.6–13.5)	4.2 (0.5–7.8)	5.9 (2.8–9.1)	7.1 (1.5–12.8)	7.1 (1.0–13.3)	7.1 (3.0–11.3)
**Obesity**	6.8 (2.7–10.9)	12.7 (6.6–18.8)	9.4 (5.9–13.0)^€^	10.1 (4.1–16.1)	2.5 (0–5.3)	5.9 (2.8–9.1)^€^	3.6 (0–7.6)	2.9 (0–6.9)	3.2 (0.4–6.1)^€^

* For the overall sample there were 37 children with stunting and wasting, and 17 children with stunting and overweight or obesity. The remaining children with stunting are also classified in the healthy weight category.

^€^Statistically significant difference across a nutritional status category for all ages by country of origin at p<0.001.

### Comparing young refugee children with US low-income children

**Fig 1:**
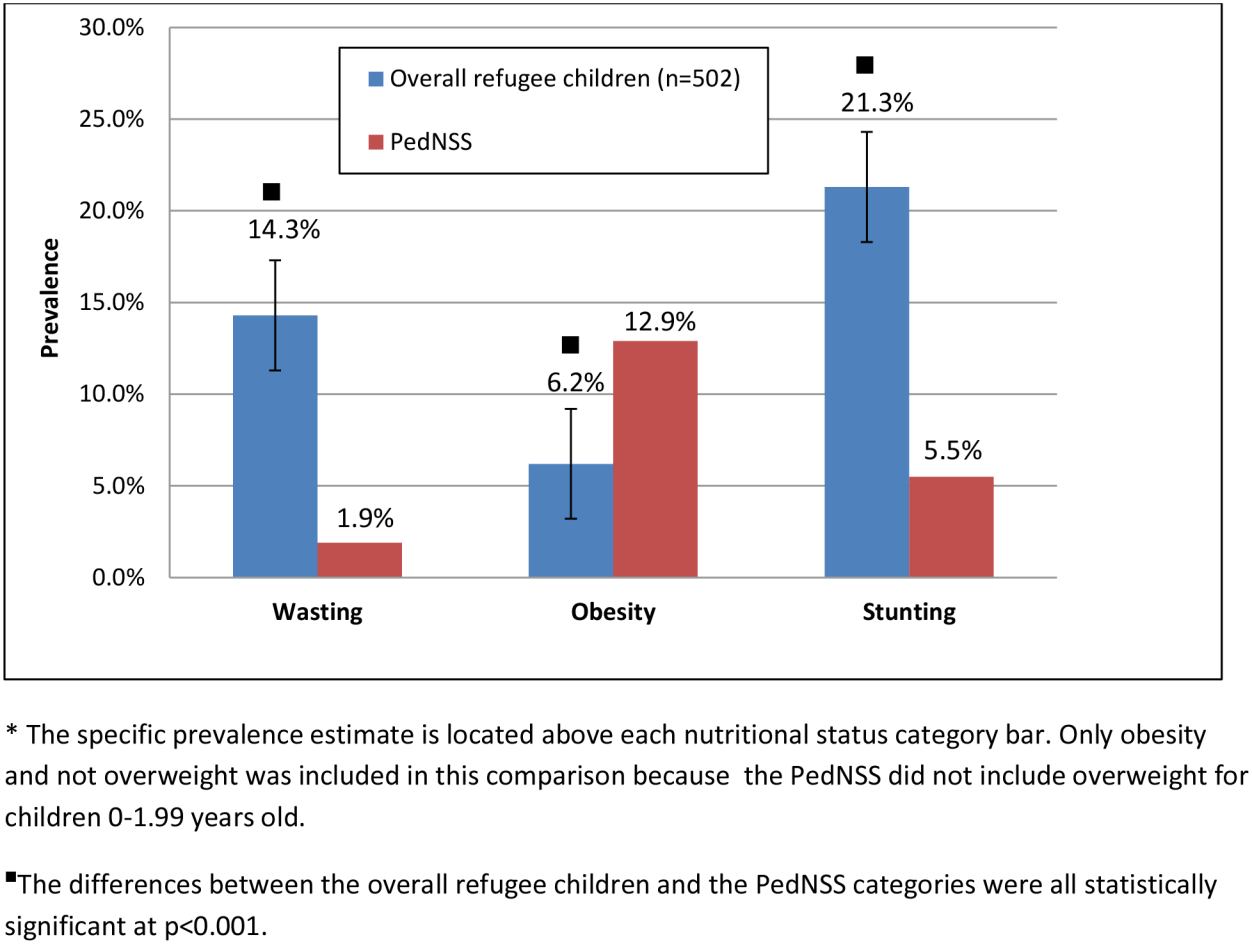
Prevalence of nutritional status categories among refugee children under 5 years old and the WA State PedNSS.

**Fig 2:**
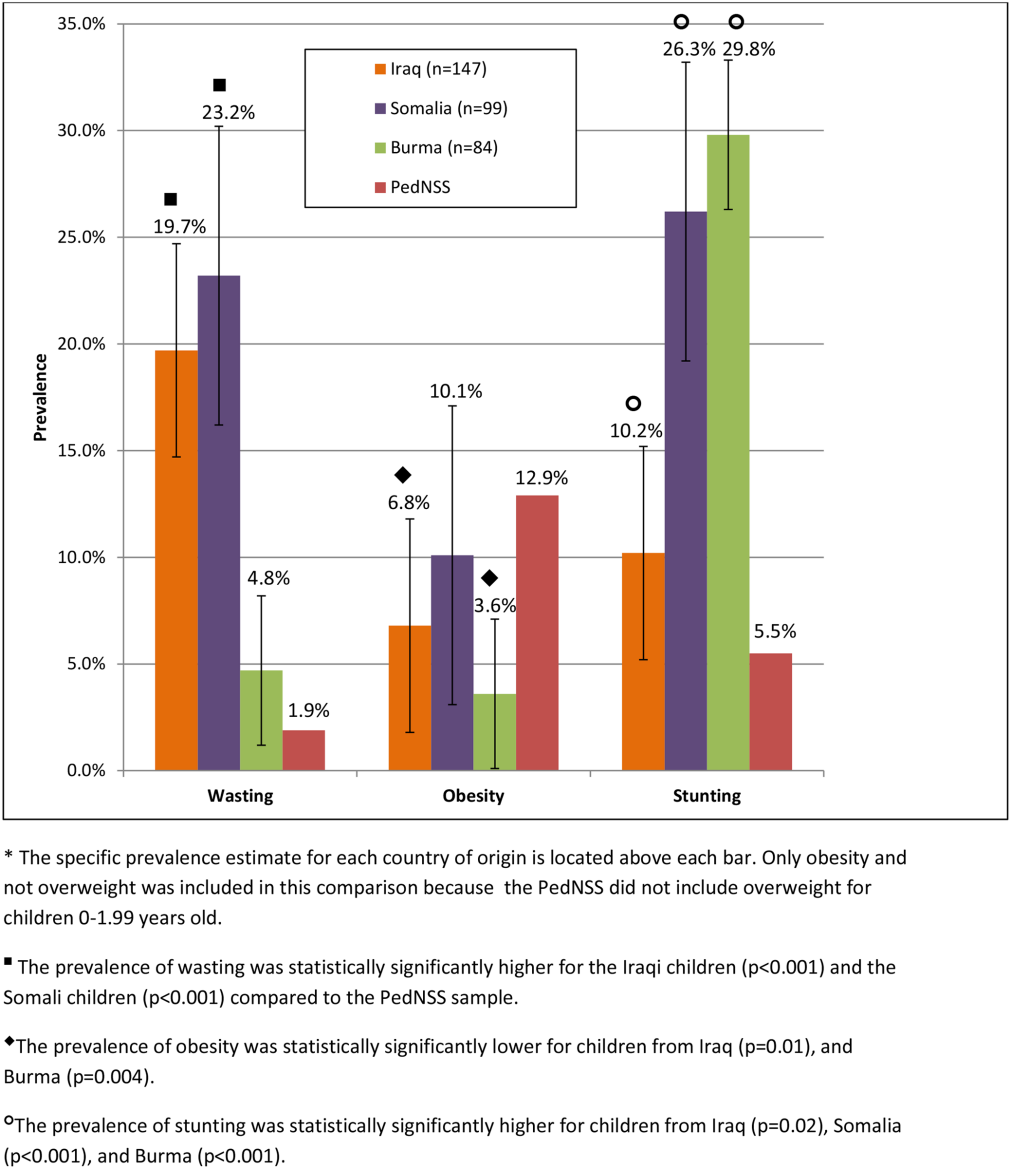
Prevalence of nutritional status categories among refugee children under 5 years old from the top three countries of origin and the WA State PedNSS.

A total of 502 refugee children under 5 years old (51.1%) were compared to the prevalence estimates from the WA PedNSS group. These young refugee children (0–5 year old) were distributed by country of origin in similar pattern as the overall group (0–10 years of age), with 65.7% from the top three countries of origin: Iraq, Somalia, and Burma. As a whole, young refugee children had a significantly higher prevalence of wasting (14.3%, p<0.001) than the WA PedNSS comparison group (1.9%; Fig 1). Wasting was significantly higher among young Somali (23.2%, p<0.001), and Iraqi children (19.7%, p<0.001) than the WA PedNSS (1.9%) (Fig 2). The overall young refugee group had a significantly higher prevalence of stunting (21.3%, p<0.001) than the WA PedNSS (5.5%). This difference was also found for the individual country of origin groups: Somali children (26.2%, p<0.001); Iraqi children (10.2%, p = 0.02), and Burmese children (29.8%, p<0.001).

All groups of young refugee children revealed some degree of overnutrition (Fig 1). For overnutrition, only obesity was compared for the young refugee children and the WA PedNSS sample because children 0–2 years old were not classified into an overweight category in the WA PedNSS sample. The prevalence of obesity for the young refugee children (6.2%, p<0.001) was significantly lower than the WA PedNSS (12.9%). The Somali children had a 10.1% prevalence of obesity, which was not statistically significantly different than the WA PedNSS (12.9%, p = 0.25). Children from both Iraq and Burma had a statistically significantly lower prevalence of obesity (6.8%, p = 0.01; 3.6%, p = 0.004, respectively) than the WA PedNSS (12.9%).

### Nutritional status of refugee children based on WHO definitions

When refugee children age 0–10 years were classified based on the WHO 2006 definitions, (S1 Table) the prevalence estimates were as follows: 8.9% for wasting, 13.2% for stunting, 6.9% for overweight and, 4.4% for obesity (S2 Table). Using the WHO 2006 definitions for children under 5 years old we found that the prevalence of wasting (7.0%, p<0.001) and stunting (15.1%, p<0.001) were significantly higher than the WA PedNSS (1.8% and 5.5%, respectively), and obesity (3.2%, p<0.001) was significantly lower (12.9%). Similar trends were noted for the top three countries of origin (S3 Table).

## Discussion

This study provides evidence of malnutrition across the nutrition spectrum for refugee children at the OME before resettlement in WA. This emerging pattern of a dual burden of under- and overnutrition among refugee children corresponds with the worldwide trend towards an increasing prevalence of overnutrition among middle and low-income countries [28]. Poor nutrition represents a significant problem in this population of refugee children, with nearly half (44.9%) found to have at least one form of malnutrition at the OME. This study also provided evidence of malnutrition among older refugee children in the 5–10 year old age group, which is a group less often discussed. The older refugee children had prevalence estimates of wasting, stunting, overweight and obesity similar to those found among the younger refugee children. Another important finding of this study was that young refugee children had a significantly higher prevalence of wasting and stunting, and a lower prevalence of obesity compared to low-income children under 5 years old in the WA PedNSS. Finally, this study provided evidence of the heterogeneity of the nutrition profile by country of origin among refugee children with significantly higher rates of obesity among the Iraqi children, wasting and stunting among the Somali children, and stunting among the Burmese children.

The prevalence of undernutrition among the refugee children in this study prior to resettlement in WA are consistent with estimates for refugees examined by the International Organization for Migration (IOM) panel physicians [9]. The 2012 IOM report of 7,439 refugee children under 5 years of old from Somalia, Ethiopia, the Democratic Republic of Congo, Eritrea, Iraq, Sudan, Burma, and Bhutan, reported a lower prevalence of wasting (7.5%) than our study (16.9%), and a comparable rate of stunting to our study (19.4% versus 20.6%). The higher prevalence of wasting in our study compared to the IOM report may be due to the difference in anthropometric definitions because the IOM report used the WHO 2006 definitions for children 0–5 years old while our study used the WHO 2006 for 0–1.99 year olds and CDC 2000 definitions for 2–5 year olds [22]. Another potential explanation for the difference in prevalence estimates is the relative proportions of the countries contributing to the total sample, as our study demonstrates significant variation in nutritional status category prevalence estimates across different countries of origin. Further, the populations included in the IOM report may have had different pre-resettlement experiences than the children in our study sample, which may have contributed to a difference in prevalence estimates.

One study that included all US-bound Iraqi refugees examined by IOM in Jordan included nutritional status estimates for both adults and children and was conducted by the CDC and IOM [29]. This study included 5,734 children ages 2–19 years old, and they subdivided children into age group categories, including 2–5 years old and 6–11 years old. The anthropometric definitions for the nutritional status categories in the CDC and IOM study were the same CDC 2000 definitions used in our study. When we compare the prevalence estimates among children 2–5 years old in the CDC and IOM study to the results for children under 5 years old in our study, the prevalence estimate for wasting was lower in the CDC and IOM study at 11.8% compared to 19.7% in our study. The prevalence estimates for overnutrition were higher in the CDC and IOM study for 2–5 years olds than the under 5 year olds in our study, (overweight: 14.6% versus 8.8%, and obesity: 14.4% versus 6.8%, respectively). In the older cohort of children 6–11 years old in the CDC and IOM study the prevalence estimates were more comparable to the 5–10 year old children in our study (wasting: 10.9% versus 12.7%, overweight: 12.9% versus 11.0%, and obesity: 9.4% versus 12.7%, respectively). The difference in prevalence estimates between the CDC and IOM study, and our study may be due to a difference in the way the age categories were subdivided, in particular because the IOM and CDC study did not include children 0–2 years old. Another contributing factor to the difference in the prevalence estimates between the two studies may be a change in the circumstances for Iraqi refugees over time as the IOM and CDC study occurred from 2007–2009 compared to 2012–2014 for our study. For example, families may have had differing levels of food security and access to therapeutic feeding programs between the two studies leading to a higher prevalence of wasting in our study. In addition, children in our study had their OME at a variety of settings including within Iraq, while the children in the IOM and CDC study all had their OME in Jordan, suggesting they may have had different pre-resettlement nutrition experiences.

There is a dearth of published studies that report both under- and over-nutrition among refugee children at their DME. The median time interval from OME to DME in our study was 5.5 months (IQR 4.3–6.5 months); therefore, similar prevalence estimates may be found when comparing malnutrition at the OME to the DME. One study evaluated the prevalence of wasting and stunting among refugee children under 5 years old overall and by country of origin at their DME in DeKalb County, Georgia (GA) from 2010–2011 [30]. This GA study found that children under 5 years old had a 12% prevalence of wasting at the DME, which was comparable to the 14.3% observed in our study. In the GA study, children from Burma had a higher prevalence of wasting at 9.5% than the children from Burma in our study (4.8%). The GA study also found a similar prevalence of stunting overall in children under 5 years old (18.4%) compared to the children in our study (21.3%). However, among children from Burma we found a slightly higher prevalence of stunting (29.8%) compared to the GA study (22.1%). The differences in prevalence estimates of malnutrition may reflect the use of the WHO 2006 definitions in the GA study for children age 2–5 years old compared to the CDC definitions utilized in our population[22]. An additional explanation for the difference in prevalence estimates between the two sites may be due to the influence of nutrition interventions that could have occurred between the OME and the DME, or due to differences in nutrition experiences before resettlement.

Most studies of refugee children have focused on young children under five years old. Our study demonstrates that school age children (5–10 years old) have similar prevalence estimates of malnutrition to younger children. The prevalence of wasting among 5-10-year old refugee children in our study was overall higher than the prevalence among children under 5 years old (20.4% versus 14.3%). By country of origin, a higher rate of wasting among older children was found for Somali and Burmese children as compared to those under 5 years old (Table 3). The prevalence of stunting among Burmese 5–10 year olds was found to be higher than the younger Burmese children (48.6% versus 29.8%), (Table 3). These differences by age group may reflect older children’s longer duration in a displaced setting, or may signify a greater prioritization placed by overseas nutrition programs on younger age groups rather than school age children. These findings support the importance of nutrition interventions for older children before and after resettlement that target the feeding practices and physical activity of school age children.

The difference in the nutritional status category prevalence estimates between children whose families originated from Iraq, Somalia and Burma is related to underlying country of origin level differences in nutritional status [31] and may be due to variation in the location and duration of time in transition from their country of origin to an eventual country of resettlement. In fact, some of the children in our sample may have never lived in their family’s country of origin. The families location and time in transition may have contributed to food insecurity, episodes of illness, and exposure to traumatic events [32], and these differences in living environments contribute to the child’s health profile [33]. For example, the increased conflict leading to a marked increase in the number of Iraqis seeking refugee status began in 2003 compared to the decades of conflict and persecution experienced by many families in Somalia and Burma. Therefore, the higher prevalence of overnutrition among Iraqi children may be due to a more recent change in food access and security, and a higher prevalence of stunting among Somali and Burmese children may be due to a prolonged period of suboptimal nutrition and illness. Future larger studies should also explore differences between refugee camps, as well as between refugee camps and secondary resettlement to another city or region, because significant heterogeneity likely exists between individuals from the same country of origin based on differences in their pre-resettlement experiences.

Several limitations in our study should be considered. First, this is a cross-sectional study at the OME, and the prevalence estimates presented represent a single time point. Second, all anthropometric measurements are based on age, and some refugee children’s birthdate (month and day) may have been unknown to the family and is therefore listed as January 1^st^ of a known year. This may lead to either over- or under-estimation of the child’s age potentially leading to misclassification of the child’s nutritional status category. Third, it is possible that the comparison group from WA PedNSS includes resettled refugee children. However, we believe that this would be an insignificant proportion of the total population and inclusion would bias our results toward the null hypotheses that there are no differences between the groups. Additionally, this study was not able to account for family level clustering of nutritional status as information regarding which children were siblings was unavailable [34].

Finally, the prevalence estimates of the nutrition status categories differed when the anthropometric definitions from the combined WHO 2006 and CDC 2000 definitions (Table 1) and the WHO 2006 definitions alone (S1 Table) were used. The CDC described this difference in a previous study, which found that the use of the combined WHO 2006 and CDC 2000 definitions leads to a higher reported prevalence of wasting than the WHO 2006 definitions alone [35]. When we compare our findings for the overall refugee group with the combined WHO 2006 and CDC 2000 definitions (Table 2) to the WHO 2006 definitions (S2 Table) we found that the prevalence estimate of wasting is 8.4% higher, stunting is 6.9% higher, overweight is 0.7% higher, and obesity is 1.5% higher. This difference in prevalence estimates based on the anthropometric definition used should be considered when comparing between studies and when evaluating standards to determine children that are at risk following resettlement in the US.

### Implications

Our findings that refugee children are affected by the dual burden of under- and over-nutrition supports the importance of addressing the entire spectrum of malnutrition when designing child nutrition programs before resettlement and targeted interventions after US resettlement. Interventions overseas should focus on all children 0–10 years old, and should address overweight prevention in addition to undernutrition in selected locations. Post-resettlement interventions should address healthy eating and active living for all ages of newly resettled children. Children with undernutrition would benefit from nutrition counseling and support from their medical provider and dieticians to achieve a healthy weight. Overweight children should also receive support for healthy weight maintenance, physical activity opportunities, and chronic disease prevention. Given that there are differences in nutritional status categories between children based on country of origin, cultural tailoring and consideration of before resettlement experiences may be included in intervention development.

The changing nature of worldwide refugee crises necessitates timely nutrition briefs based on country of origin (and sub-divided by ethnic groups as data is available) spanning the nutrition spectrum, and including multiple age groups. This study supports the need for more longitudinal studies following refugee children before and after resettlement in order to delineate their recovery from wasting and stunting, and their risk for overweight development and obesity-associated chronic diseases. Additional studies about refugee children may help public health practitioners, physicians, dieticians, and community organizations develop effective, culturally tailored interventions to decrease the risk of poor health outcomes and promote healthy growth.

## Supporting Information

S1 Table Nutritional status categories of refugee children based on the WHO 2006 definitions. (DOCX)

S2 Table Prevalence estimates for the nutritional status categories based on WHO definitions for refugee children at the overseas screening medical examination overall, % (95% CI). (DOCX)

S3 Table Prevalence estimates for the nutritional status categories based on WHO definitions for refugee children at the overseas screening medical examination by country of origin, % (95% CI). (DOCX)
