# Supplementary material for: Comparison of the Nutritional Status of Overseas Refugee Children with Low Income Children in Washington State
Source: PLoS One. 2016 Jan 25;11(1):e0147854. doi: 10.1371/journal.pone.0147854 (PMC4725764; doi:10.1371/journal.pone.0147854)
Supplement: S1 Table — (DOCX) [file pone.0147854.s001.docx]

**Supporting Information**

**S1 Table. Nutritional status categories of refugee children based on the WHO 2006 definitions.**

| **Nutritional status category** | **Age 0-5 years^∫^** | **5 – 10 years^∫^** |
| --- | --- | --- |
| **Stunting*** | Height/length-for-age ≤ 2.3^rd^ percentile | |
| **Wasting** | Weight-for-length ≤ 2.3^rd^ percentile | BMI ≤ 2.3^rd^ |
| **Healthy weight** | Weight-for-length > 2.3^rd^ percentile and ≤ 97.7^th^ percentile | BMI > 2.3^rd^ percentile and ≤ 97.7^th^ percentile |
| **Overweight** | Weight-for-length > 97.7^th^ percentile and < 99.8^th^ percentile | BMI > 85^th^ percentile and < 97.7^th^ percentile |
| **Obesity** | Weight-for-length ≥ 99.8^th^ percentile | BMI ≥ 97.7^th^ percentile |

The 0.2^nd^ percentile is equal to z-score of -3.00, 2.3^rd^ percentile is equal to z-score of -2.00, 97.7^th^ percentile is equal to a z-score of 2.00 and 99.8^th^ percentile is qual to a z-score of 3.00.

**^∫^** Based on WHO 2006 definitions.[[20](#_ENREF_20)]

*Children classified with stunting will also be classified into one of the four weight-for-height categories.
