# Supplementary material for: Comparison of the Nutritional Status of Overseas Refugee Children with Low Income Children in Washington State
Source: PLoS One. 2016 Jan 25;11(1):e0147854. doi: 10.1371/journal.pone.0147854 (PMC4725764; doi:10.1371/journal.pone.0147854)
Supplement: S2 Table — (DOCX) [file pone.0147854.s002.docx]

**Supporting Information**

**S2 Table**. Prevalence estimates for the nutritional status categories based on WHO definitions for refugee children at the overseas screening medical examination overall, % (95% CI).

| **Nutritional status category** | **All Countries** | | |
| --- | --- | --- | --- |
|  | **<5 years** | **5-10 years** | **All Ages** |
|  | **n=502** | **n=480** | **n=982** |
| **Stunting*** | 15.1 (12.0-18.3) | 11.3 (8.4-14.1) | 13.2 (11.1-15.3) |
| **Wasting** | 7.0 (4.7-9.2) | 10.8 (8.0-13.6) | 8.9 (7.1-10.6) |
| **Healthy weight** | 84.7 (81.5-87.8) | 74.8 (70.9-78.7) | 79.8 (77.3-82.4) |
| **Overweight** | 5.2 (3.2-7.1) | 8.8 (6.2-11.3) | 6.9 (5.3-8.5) |
| **Obesity** | 3.2 (4.7-9.2) | 5.6 (3.6-7.7) | 4.4 (3.1-5.7) |
