# Supplementary material for: Comparison of the Nutritional Status of Overseas Refugee Children with Low Income Children in Washington State
Source: PLoS One. 2016 Jan 25;11(1):e0147854. doi: 10.1371/journal.pone.0147854 (PMC4725764; doi:10.1371/journal.pone.0147854)
Supplement: S3 Table — (DOCX) [file pone.0147854.s003.docx]

**Supporting Information**

**S3 Table**. Prevalence estimates for the nutritional status categories based on WHO definitions for refugee children at the overseas screening medical examination by country of origin, % (95% CI).

| **Nutritional status category** | **Iraq** | | | **Somalia** | | | **Burma** | | |
| --- | --- | --- | --- | --- | --- | --- | --- | --- | --- |
|  | **< 5 years** | **5-10 years** | **All ages** | **< 5 years** | **5-10 years** | **All ages** | **< 5 years** | **5-10 years** | **All ages** |
|  | **n=147** | **n=118** | **n=265** | **n=99** | **n=120** | **n=219** | **n=84** | **n=70** | **n=154** |
| **Stunting*** | 6.8 (2.7-10.9) | 3.4 (0-6.7) | 5.3 (2.6-8.0) ^€^ | 21.2 (12.9-29.4) | 7.5 (2.7-12.3) | 13.7 (9.1-18.3) ^€^ | 22.6 (13.8-31.8) | 34.3 (22.9-45.7) | 27.9 (20.8-35.1) ^€^ |
| **Wasting** | 10.9 (5.8- 16.0) | 6.8 (2.2-11.4) | 9.1 (5.6-12.5) ^€^ | 12.1 (5.6-18.7) | 19.2 (12.0-26.3) | 16.0 (11.1-20.9) ^€^ | 1.2 (0-3.6) | 4.3 (0-9.1) | 2.6 (0-5.1) ^€^ |
| **Healthy weight** | 79.6 (73.0-86.2) | 66.1 (57.4-74.8) | 73.6 (68.2-78.9) | 76.8 (68.3-85.2) | 71.7 (63.5-79.8) | 74.0 (68.1-79.8) | 91.7 (85.6-97.7) | 85.7 (77.3-94.1) | 89.0 (84.0-94.0) |
| **Overweight** | 5.4 (1.7-9.2) | 13.6 (7.3-19.8) | 9.1 (5.6-12.5) | 6.1 (1.3-10.8) | 6.7 (2.1-11.2) | 6.4 (3.1-9.7) | 4.8 (0-9.4) | 7.1 (1.0-13.3) | 5.8 (2.1-9.6) |
| **Obesity** | 4.1 (0.8-7.3) | 13.6 (7.3-19.8) | 8.3 (5.0-11.6) ^¥^ | 5.1 (0.7-9.4) | 2.5 (0-5.3) | 3.7 (1.1-6.2) ^¥^ | 2.4 (0-5.7) | 2.9 (0-6.9) | 2.6 (0-5.1) ^¥^ |

*For the overall sample there were 12 children with stunting and wasting, and 6 children with stunting and overweight or obesity, the remaining children with stunting were in the healthy weight category

^€^Statistically significant difference across a nutritional status category for all ages by country of origin at p<0.001

^¥^ Statistically significant difference across a nutritional status category for all ages by country of origin at p<0.05
